# Destruction and regeneration in bone and cartilage

**DOI:** 10.1186/s41232-023-00313-2

**Published:** 2023-12-13

**Authors:** Noriyuki Tsumaki

**Affiliations:** 1 Department of Tissue Biochemistry, Graduate School of Medicine, Osaka University, Osaka, Japan; 2 Department of Tissue Biochemistry, Graduate School of Frontier Biosciences, Osaka University, Osaka, Japan; 3 Premium Research Institute for Human Metaverse Medicine (WPI-PRIMe), Osaka University, Osaka, Japan

Bone and cartilage compose the skeleton that sustains the body of vertebrates, including humans, and are indispensable tissues for motion. Diseases of the bone and cartilage impair their function of sustaining the body and moving the joints, negatively impacting an individual’s quality of life over a prolonged duration of time. Arthritis affects bone and cartilage and is one of the leading conditions that hinder the independent living of individuals [1]. Bone and cartilage are closely related tissues because most bones are formed through a process known as endochondral bone formation; in this process, cartilage primordia, which is initially formed during development, is subsequently replaced by bone at the center of the primordia while the cartilage at the ends persists as articular cartilage [2]. After birth, each bone and cartilage also performs specific functions. Bone undergoes continuous remodeling, via bone-resorbing osteoclasts and bone-forming osteoblasts, to obtain appropriate shapes for healthy skeletal structure and motion. Articular cartilage comprises chondrocytes embedded in an abundant extracellular matrix that confer the mechanical function of lubrication and shock absorption in joints. Recent progress in the field of skeletal research has identified subtypes of cells that contribute to homeostasis and the pathology of bone and cartilage. To develop treatments for diseases of the bone and cartilage, a precise understanding of the biology and pathology of tissues is needed.

In this thematic series of reviews, we invited the leading researchers working in the field of skeletal research. Dr. Saito summarized the regulation and function of the superficial zone of articular cartilage, which contains abundant proteoglycan 4 (PRG4). This proteoglycan, also known as lubricin, is known to play important roles in joint lubrication. Recent studies on the function of PRG4 as a signaling molecule as well as the regulation of PRG4 and the superficial zone were reviewed. In addition, drug-discovery research for osteoarthritis involving the targeting of PRG4 is discussed. Dr. Agemura and colleagues reviewed their series of original studies identifying arthritis-associated osteoclastogenic macrophages (AtoMs) that give way to pathological osteoclasts under inflamed conditions. Pathological osteoclasts are distinct from those present under physiological conditions. In addition, an in vivo imaging technique using two-photon microscopy is explained.

We would like to express our sincere gratitude to the distinguished researchers who contributed to this series of reviews and sincerely hope these articles provide novel insights to researchers in the broad field of inflammation and regeneration.
